# Toothbrush Resistance of Resin-Based Stain and Glaze Materials Applied to 3D-Printed Denture Resins

**DOI:** 10.3390/ma19112190

**Published:** 2026-05-22

**Authors:** Panisa Homyai, Ting-Chia Liu, Princy Thakkar, Chin-Chuan Fu, Nathaniel C. Lawson, Rama Kiran Chavali

**Affiliations:** 1 Department of Restorative Sciences, School of Dentistry, University of Alabama at Birmingham, Birmingham, AL 35294, USA; phomyai@uab.edu (P.H.); ccfu@uab.edu (C.-C.F.); 2 Department of Clinical and Community Sciences, School of Dentistry, University of Alabama at Birmingham, Birmingham, AL 35294, USA; tliu@uab.edu (T.-C.L.); thakkarp@uab.edu (P.T.); nlawson@uab.edu (N.C.L.)

**Keywords:** 3D printing, denture base resin, surface glaze, toothbrushing abrasion, surface gloss

## Abstract

Three-dimensional (3D)-printed dentures are often fabricated from a single tooth-colored resin and externally characterized using stains and glaze coatings to enhance gingival esthetics and surface properties. However, routine toothbrushing may degrade these coatings, potentially affecting surface gloss and roughness. This study evaluated the effects of stain timing and glaze application on the gloss and surface roughness of a 3D-printed denture resin following simulated toothbrushing. Eighty disc-shaped specimens (12 mm × 3 mm) were fabricated and assigned to two staining systems (OPTIGLAZE Color and Palette 2.0), with subgroups based on stain timing (before or after post-curing) and glaze application (with or without glaze) (*n* = 10). Specimens underwent 20,000 cycles of simulated toothbrushing, and gloss and surface roughness were measured before and after brushing. Data were analyzed using two-way ANOVA (α = 0.05). Glaze application significantly improved gloss retention for both staining systems (*p* < 0.001), while stain timing had no independent effect. Glaze application with Palette 2.0 demonstrated improved gloss retention when post-cured in a post-curing unit. Toothbrushing increased surface roughness in all groups, with no significant effects of stain timing or glaze. Within the limitations of this study, glaze improves gloss stability, whereas stain timing has minimal influence and does not affect surface roughness.

## 1. Introduction

Three-dimensional (3D) printing technologies have expanded the range of workflows for complete denture fabrication. Contemporary printed dentures may be produced either by printing the denture base and teeth as a single monolithic prosthesis or by fabricating the teeth and denture base separately and subsequently bonding the components together [1]. The monolithic workflow eliminates the need for a bonding interface between denture teeth and the base and therefore reduces the potential for tooth debonding or occlusal discrepancies that may arise during assembly procedures [2,3]. In many clinical workflows, monolithic dentures produced with vat photopolymerization printers are fabricated using a single tooth-colored resin, after which external characterization is applied to replicate gingival coloration and enhance the esthetic appearance of the prosthesis [1]. Gingival characterization may be created by applying pigmented composite resins or light-cured staining materials to the printed surface of the denture. These materials may be bonded to printed denture resins using surface treatments similar to those used for printed crowns, such as airborne particle abrasion of the final cured prosthesis [4]. Alternatively, some workflows involve applying characterization materials directly to the printed surface prior to post-curing, allowing the materials to co-polymerize during final light polymerization [5]. Although this latter approach may simplify the laboratory procedure, the durability of such bonds has not been fully established.

In addition to characterization stains, protective glaze coatings are frequently applied to printed dentures to seal the surface and enhance esthetic properties [6,7,8]. Light-cured glaze systems containing nanoparticle fillers have been introduced for this purpose and are marketed for use on acrylic dentures, composite restorations, provisional prostheses, and hybrid ceramic materials. Application of these coatings has been reported to improve surface smoothness, increase hardness, increase strength, and reduce water sorption and solubility [6,7,8,9]. Surface coatings may also reduce microbial colonization and improve resistance to discoloration [7], thereby contributing to improved clinical longevity of characterized prostheses. Several commercial stain and glaze systems are currently available for use with printed prostheses, including OPTIGLAZE Color (GC Corp., Kasugai, Aichi, Japan), Palette 2.0 (Pac-Dent Inc., Anaheim, CA, USA), Rodin Glaze N2-Free (Pac-Dent Inc.) and other light-cured resin characterization materials. OPTIGLAZE Color is a liquid stain composed primarily of polymethyl methacrylate, methyl methacrylate, silica fillers, and photoinitiators, and is provided with an associated Clear glaze [10]. Palette 2.0 is a flowable paste methacrylate-based staining system that is associated with Rodin Glaze N2-Free, a methacrylate-based liquid glaze [11]. Both materials are commonly used to provide surface characterization and to enhance the optical properties of prosthetic restorations.

Despite their esthetic benefits, the long-term stability of surface stains and glaze coatings remains an important concern. A common application of printed denture materials is for provisional implant-supported fixed full-arch prostheses. For these applications, oral hygiene of the soft tissue at the interface with the prosthesis requires routine toothbrushing. This toothbrushing exposes the gingival stain and glaze to repeated mechanical abrasion that may gradually degrade the surface coating. Abrasive wear may increase surface roughness and reduce surface gloss, potentially leading to discoloration, increased plaque retention, and deterioration of esthetic appearance. Some studies have reported that the use of surface stain and glaze maintains gloss, reduces surface roughness, provides greater color stability, and results in less surface degradation after toothbrushing compared to the untreated surfaces of 3D-printed specimens [12,13,14].

Manufacturers of stain and glaze systems provide different recommendations regarding the timing of stain application and the use of protective glaze layers. Some protocols involve applying stains after complete polymerization of the printed resin, whereas others recommend applying stains prior to post-curing to allow co-polymerization with the printed material [5]. Similarly, placement of a protective glaze layer over the stain is an optional step.

To date, no studies have specifically evaluated the effect of applying stains or glazes to 3D-printed materials in either cured or uncured states [15]. Therefore, the purpose of this study was to evaluate the effect of stain application timing and glaze application on the surface gloss and roughness of a 3D-printed denture resin before and after simulated toothbrushing. This information is intended to guide optimal fabrication protocols for monolithic 3D-printed denture prostheses subjected to toothbrushing wear, such as provisional implant-supported fixed full-arch prostheses. It was hypothesized that neither the timing of stain application nor the presence of a glaze layer would significantly influence changes in gloss or surface roughness following simulated toothbrushing. The null hypotheses of this study were that (1) neither the timing of stain application nor the presence of a glaze layer would significantly influence changes in gloss following simulated toothbrushing and (2) neither the timing of stain application nor the presence of a glaze layer would significantly influence changes in surface roughness following simulated toothbrushing.

## 2. Materials and Methods

A sample size calculation (α = 0.05, Cohen’s f = 0.45, Power = 0.8) was performed using statistical software (G*Power v. 3.1.9.4; Heinrich-Heine-Universität Düsseldorf, Germany), which indicated that a minimum of 10 specimens per experimental condition was required [16]. A total of 80 disc-shaped specimens measuring 12 mm in diameter and 3 mm in thickness were designed using computer-aided design software (Autodesk Tinkercad; Autodesk Inc., San Francisco, CA, USA, www.tinkercad.com, accessed on 1 January 2026). The specimens were fabricated using a vat photopolymerization 3D printer (PRO 20; Rapid Shape GmbH, Heimsheim, Germany) with a tooth-colored resin indicated for use for prosthetic devices (Rodin Rapid Ceram; Pac-Dent). Printing was performed with a layer thickness of 100 µm according to the manufacturer’s recommendations. The disc surfaces were oriented parallel to the build platform (0° orientation) to minimize the presence of support structures on the test surface and to simulate the orientation commonly used for the external surfaces of printed dental prostheses. All stains were applied to the side of the specimen that did not contain support structures. No polishing was performed in order to maintain the as-printed surface.

To determine the appropriate method for polymerization of the stain and glaze materials, Fourier transform infrared (FTIR) spectroscopy was performed on uncured and light-cured specimens using an ATR-FTIR spectrophotometer (Alpha II ATR-FTIR; Bruker, Billerica, MA, USA). Spectra were collected from 400–4000 cm^−1^ at 4 cm^−1^ resolution. Uncured materials were placed directly onto the ATR crystal, while cured specimens were polymerized on a glass slide using either a VALO Grand curing light (Ultradent Products Inc., South Jordan, UT, USA), an Elipar S10 curing light (3M ESPE, St. Paul, MN, USA), or a Curie Plus post-curing unit (Ackuretta Technologies Inc.; Taipei, Taiwan) and transferred to the ATR crystal. Three specimens were analyzed for each condition. Spectra were baseline corrected and normalized using OPUS software. Degree of polymerization was assessed by comparing reduction of methacrylate carbon–carbon double bond peaks in cured materials relative to uncured controls.

**Figure 1:**
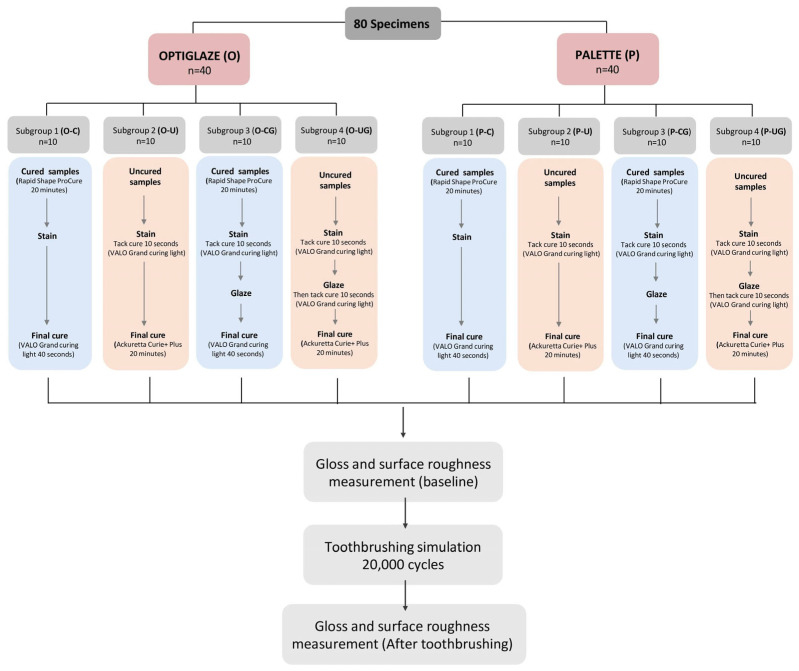
Specimen Preparation (O-C = OPTIGLAZE cured; O-U = OPTIGLAZE uncured; O-CG = OPTIGLAZE cured with glaze; O-UG = OPTIGLAZE uncured with glaze; P-C = Palette cured; P-U = Palette uncured; P-CG = Palette cured with glaze; P-UG = Palette uncured with glaze).

Specimens were divided into eight groups (*n* = 10) based on stain system (OPTIGLAZE [O] or Palette [P]), stain timing, and glaze application. In the group nomenclature, “C” denotes stain applied after post-curing (i.e., on a fully cured surface), whereas “U” denotes stain applied before post-curing (i.e., on an uncured surface). The suffix “G” indicates the presence of a glaze layer (OPTIGLAZE Clear or Rodin Glaze N2-Free). Accordingly, the groups were O-C, O-U, O-CG, O-UG, P-C, P-U, P-CG, and P-UG, as illustrated in Figure 1.

For groups in which stain was applied to cured surfaces (O-C, O-CG, P-C, P-CG), specimens were first fully polymerized (Rapid Shape Pro Cure; Rapid Shape GmbH), followed by surface cleaning with isopropyl alcohol prior to stain application. Specimens assigned to receive glaze (O-CG, P-CG) were tack-cured after staining (10 s), coated with glaze, and then final-cured (40 s), whereas non-glazed groups (O-C, P-C) were directly final-cured (40 s) after stain application. All curing of stains and glazes in these groups was performed with a dental curing light (VALO Grand; output > 1200 mW/cm^2^).

For groups in which stain was applied to uncured surfaces prior to post-curing (O-U, O-UG, P-U, P-UG), the stain (and glaze, when applicable) was applied to the printed surface, followed by tack-curing (10 s, VALO Grand). These specimens were then subjected to final polymerization in a post-curing unit approved for use with both stain materials, as well as the tooth-colored resin (Curie Plus; Ackuretta Technologies Inc.).

When applying the stain, the OPTIGLAZE material was applied with a brush, and the viscosity of the material allowed even application of a thin layer by allowing the material to self-spread. The Palette 2.0 material was thicker, and in order to allow a uniform thin layer, the material was applied to the surface of the specimens and a Mylar sheet was pressed against the specimen to obtain a uniform thickness of the material. The red OPTIGLAZE shade and the violet Palette 2.0 shade were used, as these colors were determined to provide a visually obvious appearance that would allow the operator to visualize even application of the stains.

**Figure 2:**
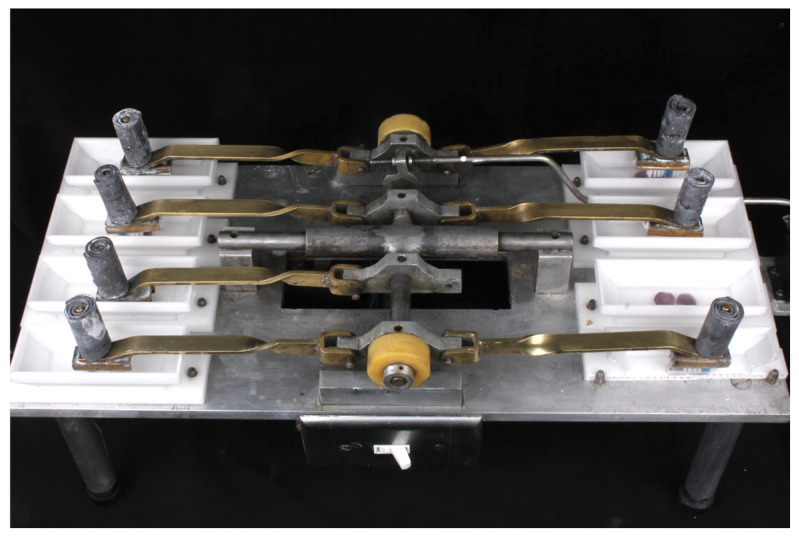
A custom toothbrushing machine. Two specimens are placed in each white tray which are then filled with dentifrice slurry (not pictured). 1 N load applied by lead weights. Horizontal movements driven by a motor.

**Figure 3:**
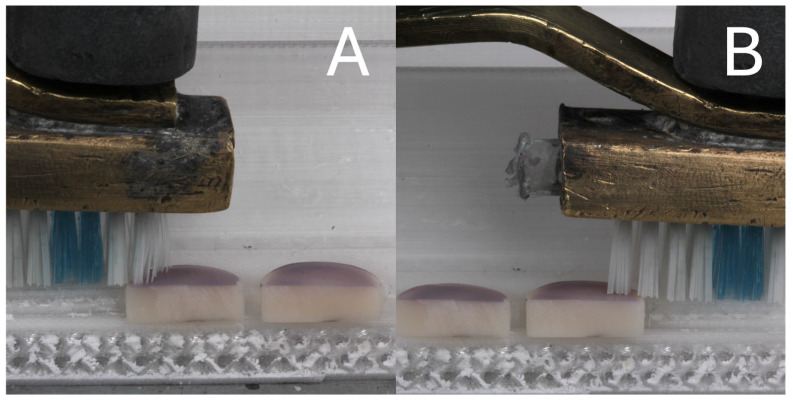
Cross-section of specimens in trays. Toothbrush head on specimen at start of cycle (**A**) and end of cycle (**B**).

Simulated toothbrushing was performed using a custom toothbrushing apparatus (Figure 2). Specimens were secured to a holder under a soft-bristled toothbrush (Oral-B; Procter & Gamble, Cincinnati, OH, USA), which was replaced after each use (Figure 3). Each brushing tray was filled with a dentifrice slurry prepared by mixing 48 g of toothpaste (Crest Pro-Health; Procter & Gamble) with 100 mL of deionized water. The specimens were brushed under a constant load of 1 N using a reciprocating horizontal motion at a frequency of 1 Hz for 20,000 brushing cycles, which is equivalent to approximately 2 years of clinical use [17,18,19]. After completion of the brushing procedure, the specimens were rinsed with distilled water for 1 min and allowed to air dry.

Surface roughness measurements were performed using a contact profilometer (SJ-210; Mitutoyo, Kawasaki, Japan) equipped with a 5 µm stylus tip and a measuring force of 4 mN following ISO 4288 [20]. An initial measurement was performed to determine the approximate arithmetic mean roughness (Ra) of each specimen. Based on the preliminary measurement, a sampling length of 4 mm with a cutoff filter of 0.8 µm was used for surfaces with Ra values greater than 0.1 µm, whereas a sampling length of 1.25 mm with a cutoff filter of 0.25 µm was used for surfaces with Ra values less than 0.1 µm. Roughness measurements were obtained at baseline and again after completion of the toothbrushing procedure. All measurements were performed by a single operator to minimize measurement variability.

Surface gloss was measured using a glossmeter (Novo-Curve; Rhopoint Instruments, St Leonards, United Kingdom) at an incidence angle of 60° with a measurement window of 4.7 × 2 mm, as previously described [21]. The device was calibrated prior to each measurement session using a black reference standard with a known gloss value. Gloss measurements were obtained at baseline and again after completion of the toothbrushing procedure. Two readings were obtained from each specimen at perpendicular orientations, and the mean value was used for statistical analysis. All measurements were performed by a single operator to ensure consistency.

Specimens from each group were sectioned in half and viewed in cross-section using a digital microscope (VHX series, Keyence Corporation, Osaka, Japan). The thickness of the stain/glaze layer was measured with internal measurement software at 10 points along the surface of the specimen (excluding the outer 1mm of the edge). The mean, minimum and maximum thickness from each specimen were recorded and averaged for each group. Additionally, surfaces of the specimens were examined with the digital microscope in order to view surface wear features.

Statistical analyses were performed using statistical software (SPSS v. 30.0.0.0; IBM Corp., Armonk, NY, USA). Data were evaluated for normal distribution and homogeneity of variance prior to statistical testing. Surface roughness and gloss were measured before and after simulated toothbrushing, and the change in each variable was calculated for each specimen to account for baseline variability and evaluate the magnitude of surface alteration caused by toothbrushing. Changes in gloss and surface roughness were analyzed separately for each stain material using two-way analysis of variance with stain application timing (before or after post-curing) and glaze application (with or without glaze) as fixed factors. Interaction effects between these variables were also assessed. Statistical significance was established at α = 0.05.

## 3. Results

### 3.1. Gloss Change

**Figure 4:**
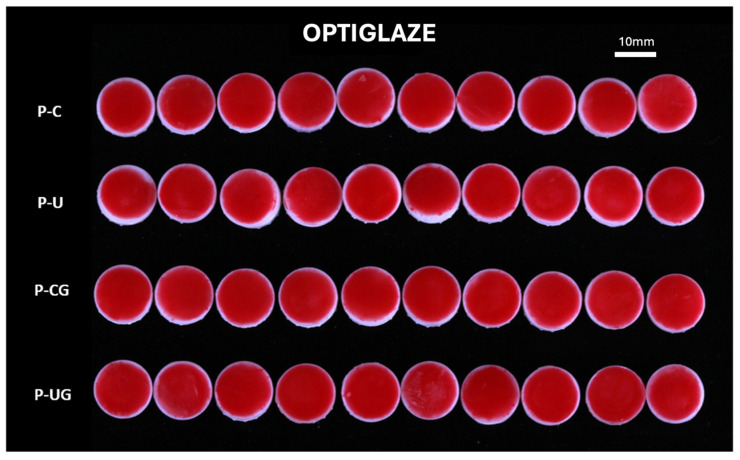
OPTIGLAZE specimens after the toothbrushing test (O-C = OPTIGLAZE cured; O-U = OPTIGLAZE uncured; O-CG = OPTIGLAZE cured with glaze; O-UG = OPTIGLAZE uncured with glaze).

**Figure 5:**
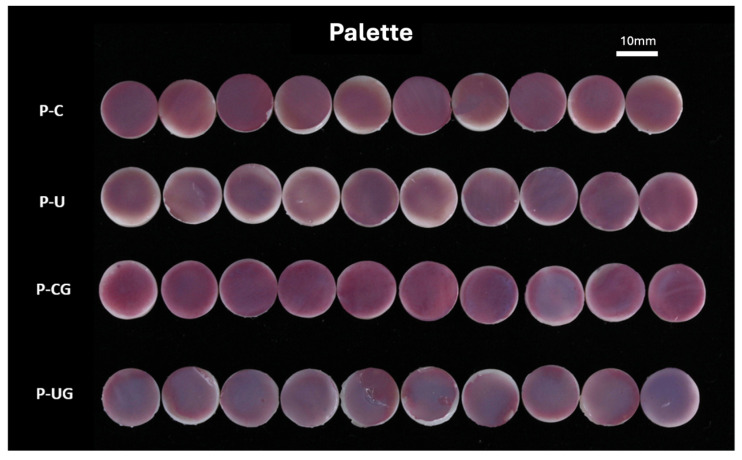
Palette specimens after the toothbrushing test (P-C = Palette cured; P-U = Palette uncured; P-CG = Palette cured with glaze; P-UG = Palette uncured with glaze).

**Figure 6:**
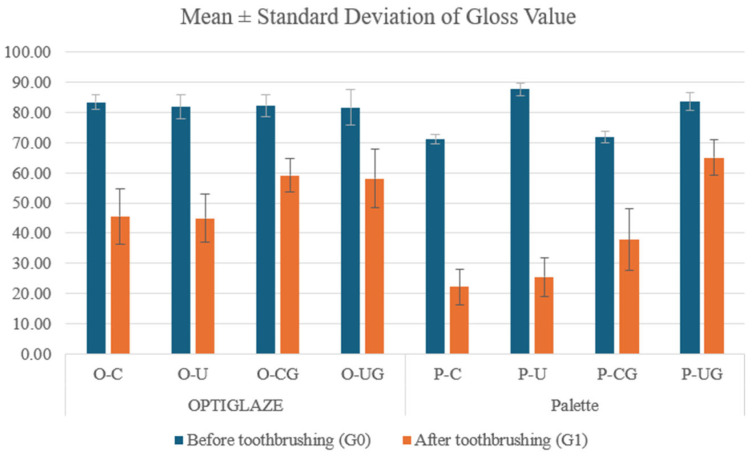
Mean ± standard deviation of gloss value (GU) of each group.

Specimens after the toothbrushing test are shown in Figure 4 and Figure 5. The means ± standard deviations of the gloss value of each group are shown in Figure 6. The two-way ANOVA results of the gloss value between stain timing and glaze are shown in Table 1.

Gloss change following toothbrushing was analyzed separately for the OPTIGLAZE and Palette stains using two-way ANOVA, with stain timing (before or after post-curing) and glaze application (with vs without glaze) as factors. For OPTIGLAZE, a significant main effect of glaze was observed (*p* < 0.001), whereas stain timing (*p* = 0.941) and the interaction between stain timing and glaze (*p* = 0.797) were not significant. For the Palette stain, a significant main effect of glaze (*p* < 0.001) and a significant interaction between stain timing and glaze (*p* < 0.001) were identified, while stain timing alone was not significant (*p* = 0.643).

### 3.2. Surface Roughness Change

**Figure 7:**
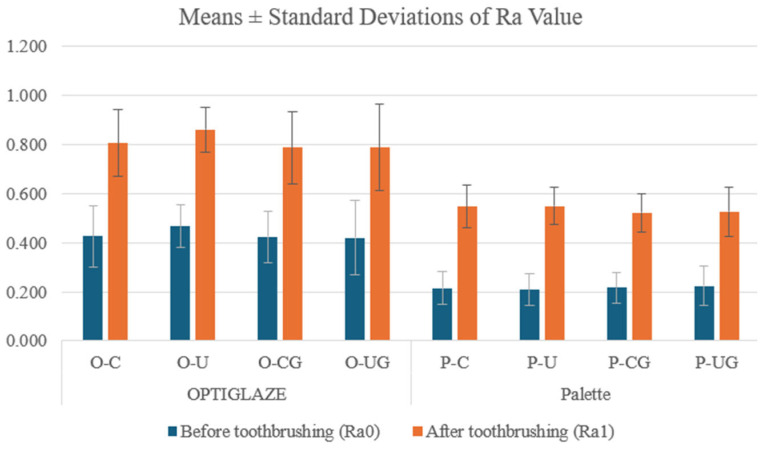
Mean ± standard deviation of Ra value (µm) of each group.

The means ± standard deviations of the Ra value of each group are shown in Figure 7. The two-way ANOVA results of the Ra value between stain timing and glaze application are presented in Table 2. Analysis of surface roughness demonstrated that toothbrushing increased roughness across all specimens; however, two-way ANOVA of roughness change (after − before toothbrushing) revealed no significant effects of stain timing, glaze application, or their interaction for either OPTIGLAZE (Timing: *p* = 0.838; Glaze: *p* = 0.629; Interaction: *p* = 0.904) or Palette (Timing: *p* = 0.945; Glaze: *p* = 0.288; Interaction: *p* = 0.829).

**Figure 8:**
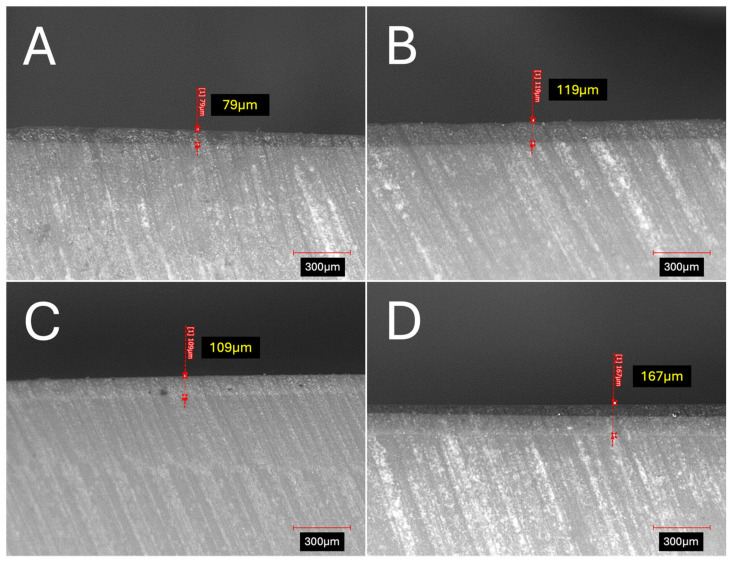
Cross-sectional digital light microscope images at 120× magnification after tooth brushing (**A**) OPTIGLAZE; (**B**) OPTIGLAZE (glazed); (**C**) Palette; (**D**) Palette (glazed).

**Figure 9:**
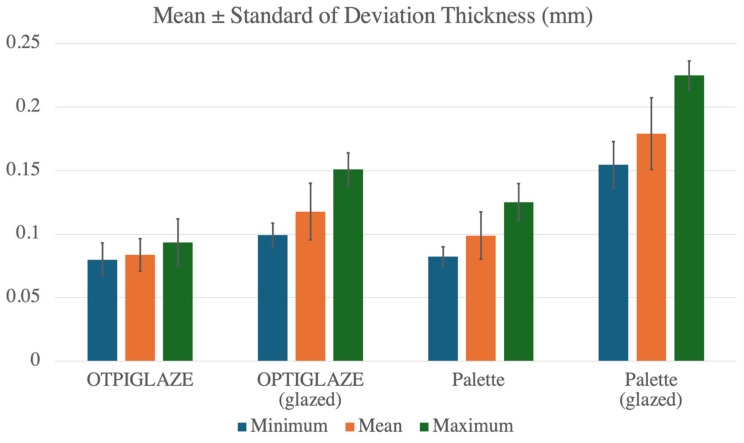
Mean ±standard deviation of thickness of stain or stain and glaze of each material.

**Figure 10:**
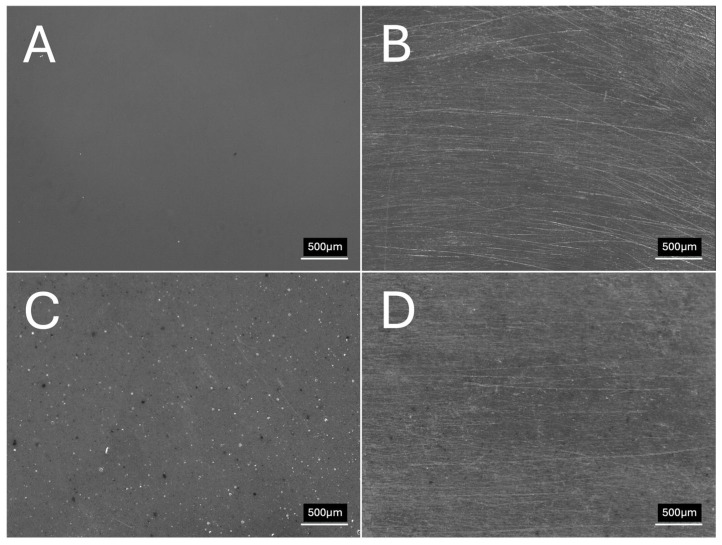
Digital light microscope images at 80× magnification (**A**) OPTIGLAZE before toothbrushing; (**B**) OPTIGLAZE after toothbrushing; (**C**) Palette before toothbrushing; (**D**) Palette after toothbrushing.

Representative images of the thickness of the stain or stain and glaze coats for both OPTIGLAZE and Palette are presented in Figure 8. The dark layer at the top of the images represents the stain/glaze layer. No difference can be visually detected between the stain and glaze layers for OPTIGLAZE. The mean, minimum, and maximum thickness of the glaze for each group are presented in Figure 9. Micrographs of the surface of the specimens before and after toothbrushing are presented in Figure 10. Representative specimens from OPTIGLAZE and Palette groups are shown to reduce redundancy, as no observable difference was noted within the subgroup.

**Figure 11:**
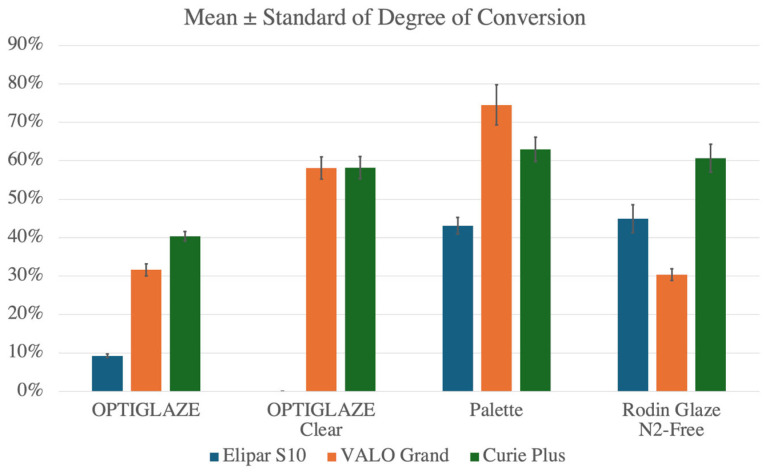
Mean ± standard deviation of degree of conversion for each stain and glaze.

The mean degree of conversion for each stain and glaze as cured by two dental curing lights and a post-curing unit are presented in Figure 11. OPTIGLAZE Clear did not achieve any polymerization with the Elipar S10 curing light.

## 4. Discussion

For gloss analysis, the two-way ANOVA showed that glaze application had a statistically significant effect on gloss values in both the OPTIGLAZE and Palette groups; however, stain timing (before or after post-curing) did not have a statistically significant effect on gloss in either group. Therefore, the null hypothesis was rejected for gloss, as glaze application significantly affected gloss values. For surface roughness, neither stain timing, glaze application, nor their interaction showed statistically significant effects in either the OPTIGLAZE or Palette groups. Therefore, the null hypothesis was accepted for surface roughness, since neither stain timing nor glaze application had a significant effect on Ra values.

Although glaze application significantly improved gloss retention, it did not significantly affect surface roughness following toothbrushing. This may be explained by the fact that gloss and surface roughness represent related but distinct surface properties. Toothbrushing produced light superficial scratches in all groups (Figure 10), resulting in measurable increases in Ra values regardless of glaze application. However, these surface defects may not have been sufficiently severe to disrupt specular light reflection when a clear glaze layer remained present. In contrast, surface abrasion directly affecting the more opaque pigmented stain layer may have increased light scattering and reduced gloss to a greater extent. Therefore, although glaze application did not prevent measurable increases in surface roughness, the transparent glaze layer may have better preserved the optical uniformity of the surface and maintained gloss following toothbrushing.

A previous study examined roughness and gloss of several glazes used for 3D-printed materials following 100,000 cycles of toothbrushing at 1.96 N and immersion in citric acid [12]. Initial values of gloss (35–70 GU) and Ra (0.2–0.7 µm) were within a similar range as the present study. In that study, only one of the three glazes tested showed a significant decrease in gloss, and none of the materials experienced a significant increase in roughness following toothbrushing [12]. None of the same materials from that study were used in the current study. In another study, the roughness of a denture base glaze following 50,000 cycles of toothbrushing increased with a similar magnitude as reported in the current study (0.25 µm to 0.43 µm); however, the magnitude of gloss reduction was much less (144 to 133 GU) [22].

The baseline gloss values of all OPTIGLAZE subgroups were above 80 GU, while the Palette subgroups ranged from approximately 71 to 88 GU, indicating high initial surface gloss. After toothbrushing, gloss values decreased in all groups, ranging from approximately 22 GU to 65 GU. Previous literature suggests that a clinically acceptable gloss value perceived by dentists for direct composite is approximately 40–50 GU, and values below 40 GU may be considered clinically unacceptable [23]. After toothbrushing, several subgroups, particularly Palette P-C, P-U, and P-CG, exhibited gloss values below 40 GU, indicating substantial gloss degradation. Although the 40 GU threshold reported for clinical acceptability of a direct composite used to restore teeth may not directly apply to the clinical application of gingival staining, the magnitude of gloss reduction reported in this study implies that these changes would be clinically observable. Additionally, these same stains are available in tooth shades which may be used for characterization of denture teeth, in which case the previously reported gloss threshold would be directly applicable.

Subgroups with glaze application demonstrated higher post-toothbrushing gloss values and less gloss reduction compared to those without glaze. This finding is supported by the two-way ANOVA results, which showed that glaze application had a statistically significant effect on gloss in both the OPTIGLAZE and Palette groups (*p* < 0.001). Cross-sectional thickness measurements (Figure 8 and Figure 9) demonstrated that glaze application increased the surface layer thickness, which may have contributed to improved resistance to gloss degradation. Glaze appeared to provide greater protection against gloss reduction, particularly when combined with post-curing stain application in the Palette group, as indicated by the significant interaction effect. The greater protection observed in the Palette post-cure group may be explained by the use of the Curie Plus post-curing unit for final polymerization of Rodin Glaze N2-Free, which achieved greater polymerization than the VALO Grand light-curing unit (Figure 10). The OPTIGLAZE Clear glaze, on the other hand, achieved similar polymerization with both the VALO Grand and Curie Plus.

Surface roughness analysis demonstrated that toothbrushing increased roughness in all subgroups of both materials. Figure 10 demonstrates that both materials experience scratching from toothbrush bristles. In the OPTIGLAZE group, mean Ra values increased from approximately 0.420–0.467 µm at baseline to 0.788–0.861 µm after toothbrushing. Similarly, in the Palette group, Ra values increased from approximately 0.209–0.225 µm to 0.523–0.550 µm after toothbrushing. However, two-way ANOVA revealed that stain timing, glaze application, and their interaction had no statistically significant effects on Ra values in all groups (all *p* > 0.05). Alsalem et al. performed a study in which 3D-printed materials were either glazed or unglazed and then subjected to toothbrushing. In their study, no significant reduction in roughness was noted following toothbrushing; however, their study applied only 0.2 N of load [14]. Bollen et al. reported that surface roughness affects bacterial plaque retention on intraoral hard surfaces and identified a threshold value of Ra = 0.2 µm, below which further reduction does not significantly decrease plaque accumulation. When surface roughness exceeds this threshold, plaque accumulation increases, thereby elevating the risk of caries and periodontal inflammation [24]. In the present study, all subgroups exhibited Ra values well above 0.2 µm after toothbrushing. These findings suggest a potential increase in plaque retention and surface discoloration regardless of glaze application. An Ra threshold of 0.5 µm has been reported for patient perceptibility with their tongue [25]. Therefore, this study reveals that all stain/glaze application protocols that are below tongue perceptibility before toothbrushing, however, exceed this roughness following toothbrushing.

For long-term esthetic outcomes, the appearance of 3D-printed restorations is influenced by gloss stability and resistance to surface degradation. Based on previous studies, 10,000 toothbrushing cycles are considered equivalent to approximately 1 year of clinical use; therefore, 20,000 cycles in this study represent approximately 2 years of simulated clinical use [17]. The cross-sectional measurements (Figure 9) and images (Figure 8) indicated that glaze application increased the thickness of the surface layer (OPTIGLAZE from 83 µm without glaze to 120 µm with glaze and Palette from 99 µm without glaze to 180 µm with glaze), which may help protect the underlying stain layer from eventual wear and may result in improved esthetic outcomes. In comparison, non-glazed specimens exhibited surface wear directly at the stain layer. Therefore, glaze application is recommended to enhance long-term esthetic stability.

Visual examination following toothbrushing demonstrated that stain remained on the specimen surfaces (Figure 4 and Figure 5). This finding contrasts with clinical anecdotes suggesting that these stains are readily removed with routine brushing. Adequate polymerization with the handheld curing light appears to be critical for stain durability. According to the manufacturers’ instructions for use, OPTIGLAZE requires curing with light at wavelengths below 430 nm, whereas Palette 2.0 requires wavelength between 385–515 nm. The VALO Grand curing light provides polywave output across approximately 395–480 nm, encompassing both violet and blue wavelength regions. In pilot testing, the use of a handheld curing light with a narrower output of 430 to 480 nm (Elipar S10) failed to adequately polymerize the OPTIGLAZE stain, allowing the stain to be wiped off. This observation was confirmed by measuring degree of conversion of the stains and glazes using FTIR using both curing lights and noting that OPTIGLAZE and its glaze only achieved 0 to 9% polymerization when using the Elipar S10 curing light (Figure 11).

Toothbrushing affects the surface characteristics of 3D-printed restorations. Several factors influence surface wear, including the Relative Dentin Abrasivity (RDA) value of the toothpaste, toothbrush bristle stiffness, applied brushing force, and the number of brushing cycles. In this study, a soft-bristled toothbrush (Oral-B, Procter & Gamble) and a commercially available abrasive dentifrice (Crest Pro-Health, Procter & Gamble) were used. Crest Pro-Health toothpaste has an RDA value of approximately 151, which is considered highly abrasive. Previous studies have shown that highly abrasive slurries, particularly when used with stiffer toothbrush bristles and greater brushing force, result in increased surface wear compared to medium- or low-abrasive dentifrices [26,27]. Hamza et al. reported that soft-bristle toothbrushes generally cause less abrasive wear than medium-bristle toothbrushes; however, even with soft bristles, dentin wear increased significantly as brushing force increased from 1 to 2 N [27]. In the present study, toothbrushing with a soft-bristled toothbrush and a highly abrasive toothpaste for 20,000 strokes under a 1 N load produced measurable increases in surface roughness and reductions in gloss. These findings suggest that even under controlled and moderate brushing force, the combination of extended brushing cycles and a highly abrasive dentifrice can significantly affect the surface integrity of 3D-printed materials.

A brushing force of 1 N was selected to represent a controlled, moderate force within the range reported for manual toothbrushing (approximately 1 to 2 N). In addition, this load was selected due to technical limitations of the toothbrushing apparatus, as higher forces could not be consistently applied without compromising the stability of the toothbrush heads during cyclic testing. However, clinical brushing forces vary among individuals and across different tooth surfaces. Previous studies have demonstrated that brushing forces are not uniformly distributed; occlusal and incisal surfaces may experience forces ranging from approximately 1.02 to 1.95 N, with many values clustering at the higher end (~1.69–1.95 N), whereas vestibular surfaces exhibit lower forces, ranging from approximately 1.06 to 1.64 N, with values clustering around ~1.2–1.5 N (mean ≈ 1.3 N) [18]. This pattern indicates that gingival/vestibular regions are subjected to comparatively lower mechanical loading during brushing. Therefore, the 1 N load used in the present study represents the lower range of clinically relevant forces, particularly for vestibular surfaces where gingival characterization stains are located.

This study has several limitations. The in vitro simulation does not fully replicate the complex conditions of the oral environment, including the oral microbiome, salivary factors, and intraoral thermal fluctuations. In addition, thermocycling was not performed and therefore the effects of thermal aging on the stain and glaze materials were not evaluated. Because only one printed denture resin material was evaluated, the findings should be generalized to other printed materials with caution. The use of a soft-bristled toothbrush in combination with a highly abrasive toothpaste for 20,000 strokes under a 1 N load may have influenced the results and may not accurately represent typical clinical brushing conditions. A greater brushing force (i.e., 2 N) may have resulted in greater gloss loss and increased roughness. Although all procedures were performed by a single operator to minimize variability, some unavoidable variability remained due to the manual nature of specimen fabrication and stain/glaze application. This may have resulted in variations in glaze thickness, as well as differences in the amount and consistency of the applied stain. Achieving the desired stain required balancing sufficient application to produce a uniform surface coating while avoiding excess that would be clinically unrealistic. Despite the limitations associated with manual application of the stain coatings, the coefficient of variation in layer thickness among groups remained below 20%, suggesting that variability was sufficiently controlled to allow detection of true differences between experimental conditions. The different application techniques used for OPTIGLAZE and Palette 2.0, including the use of a Mylar strip with Palette 2.0, may also have influenced specimen surface characteristics in addition to affecting stain thickness. Additional variables that could be evaluated in future studies include surface roughening (i.e., airborne particle abrasion) or smoothing (i.e., polishing after support removal) prior to stain application, as well as final curing under oxygen-inhibited reducing conditions (i.e., glycerin, nitrogen, or vacuum). Future studies should also include color stability analysis to further evaluate the long-term esthetic performance of these materials.

## 5. Conclusions

Within the limitations of this in vitro study, the following conclusions were drawn:

1. Simulated 2 years of toothbrushing increased surface roughness and reduced gloss of a 3D-printed denture resin.
2. Glaze application may protect the surface of 3D-printed denture resins from loss of gloss from toothbrushing.
3. Stain timing (before or after post-curing) appears to have minimal effect on maintenance of gloss or surface smoothness following toothbrushing. Curing of the Rodin Glaze N2-Free in a post-curing unit on uncured material maintains gloss better than curing with a handheld curing light on a cured material.
4. Stain can remain on the surface of 3D-printed denture resins after simulated 2 years of brushing when manufacturer’s recommendations are followed.

## Figures and Tables

**Table 1:** The two-way ANOVA results of the gloss value between stain timing and glaze application.

Two-Way ANOVA Results of the Gloss Value	*p* Value	
	OPTIGLAZE	Palette
Stain timing (before or after post-curing)	0.941	0.643
glaze application (with vs. without glaze)	<0.001	<0.001
Interaction between stain timing and glaze application	0.797	<0.001

**Table 2:** The two-way ANOVA results of the Ra value between stain timing and glaze application.

Two-Way ANOVA Results of the Ra Value	*p* Value	
	OPTIGLAZE	Palette
Stain timing (before or after post-curing)	0.838	0.945
glaze application (with vs. without glaze)	0.629	0.288
Interaction between stain timing and glaze application	0.904	0.829

## Data Availability

Raw data may be obtained from the corresponding author at https://drnatelawson.com/glazedata, accessed on 20 April 2026.

## Abbreviations

The following abbreviations are used in this manuscript:

3D	Three-dimensional
Ra	Arithmetic mean surface roughness
GU	Gloss units
